# The Functional Role of Spontaneously Opening GABA_A_ Receptors in Neural Transmission

**DOI:** 10.3389/fnmol.2019.00072

**Published:** 2019-03-28

**Authors:** Nathanael O’Neill, Sergiy Sylantyev

**Affiliations:** Center for Clinical Brain Sciences, University of Edinburgh, Edinburgh, United Kingdom

**Keywords:** GABA-A receptor, GABA-independent inhibition, phasic conductance, tonic conductance, G-proteins

## Abstract

Ionotropic type of γ-aminobutyric acid receptors (GABA_A_Rs) produce two forms of inhibitory signaling: phasic inhibition generated by rapid efflux of neurotransmitter GABA into the synaptic cleft with subsequent binding to GABA_A_Rs, and tonic inhibition generated by persistent activation of extrasynaptic and/or perisynaptic GABA_A_Rs by GABA continuously present in the extracellular space. It is widely accepted that phasic and tonic GABAergic inhibition is mediated by receptor groups of distinct subunit composition and modulated by different cytoplasmic mechanisms. Recently, however, it has been demonstrated that spontaneously opening GABA_A_Rs (s-GABA_A_Rs), which do not need GABA binding to enter an active state, make a significant input into tonic inhibitory signaling. Due to GABA-independent action mode, s-GABA_A_Rs promise new safer options for therapy of neural disorders (such as epilepsy) devoid of side effects connected to abnormal fluctuations of GABA concentration in the brain. However, despite the potentially important role of s-GABA_A_Rs in neural signaling, they still remain out of focus of neuroscience studies, to a large extent due to technical difficulties in their experimental research. Here, we summarize present data on s-GABA_A_Rs functional properties and experimental approaches that allow isolation of s-GABA_A_Rs effects from those of conventional (GABA-dependent) GABA_A_Rs.

## Introduction

Ionotropic receptors of γ-aminobutyric acid (GABA receptors of type A, GABA_A_Rs) are the main receptor type that generates inhibitory interneuronal signaling in the brain. The classical form of GABA_A_R-induced inhibitory signal is phasic inhibition: a short synchronized opening of GABA_A_Rs in a synapse, generated by the binding of GABA released from a presynaptic terminal. However, there is an alternative form of inhibition: charge transfer through continuously active GABA_A_Rs, or tonic inhibition, detected in peripheral nervous system in the 1970s (Brown, 1979) but documented for the central nervous system only in the 1990s (Otis et al., 1991; Brickley et al., 1996). The classical view is that tonic inhibition is generated in response to GABA, which is continuously present in the extracellular space of neural tissue due to spillover from synapses or release from astroglia and/or neurogliaform cells (Farrant and Nusser, 2005; Kozlov et al., 2006; Oláh et al., 2009). This implies the generation of a continuous inhibitory tone mainly by perisynaptic and extrasynaptic GABA_A_Rs, since the vast majority of transporters which perform reverse uptake of GABA are localized in synapses or in their immediate vicinity (Minelli et al., 1996; Chiu et al., 2002; Conti et al., 2004). Hence, the magnitude of tonic GABA_A_Rs-delivered current is considered to be regulated by the availability of extracellular GABA, and by the quantity of GABA_A_Rs at an extrasynaptic surface of a given neuron (Glykys and Mody, 2007). Later research, however, revealed that a significant part of tonic inhibition mediated by GABA_A_Rs is independent of GABA binding, i.e., it is delivered by spontaneously opening GABA_A_Rs (s-GABA_A_Rs). s-GABA_A_Rs in that study were shown to be insensitive to the competitive GABA antagonist SR-95531 (SR), but could be suppressed by the GABA_A_R open channel blocker picrotoxin (PTX), and, to the less extent, by competitive GABA antagonist bicuculline (BIC; McCartney et al., 2007).

In the last few decades, studies of GABA_A_Rs-mediated tonic currents have attracted a considerable interest, and have described a functional role of this form of inhibition in a number of brain areas; in particular, its important input into neural excitability, synaptic plasticity, neurogenesis and network oscillations (Mody and Pearce, 2004; Farrant and Nusser, 2005; Glykys and Mody, 2007). Since our understanding of underlying mechanisms is still far from excellent, the newly discovered type of tonic conductance delivered *via* s-GABA_A_Rs promises a conceptual breakthrough in the field. Nevertheless, despite the phenomenon of GABA-independent gating of GABA_A_Rs being reported in numerous publications (Neelands et al., 1999; Birnir et al., 2000; Maksay et al., 2003; Miko et al., 2004), until recently the functional role of s-GABA_A_Rs in living neural tissue has remained beyond the focus of neuroscience research.

In this article, we try to summarize the data available to date on s-GABA_A_Rs function in neural transmission and to discuss perspective directions for further studies which should clarify the role of s-GABA_A_Rs under normal conditions and in pathology.

## Functional Properties of s-GABARs

### s-GABARs: Problem of the Isolation of GABA-Independent Effects

One of the main factors which prevent a detailed study of s-GABA_A_Rs functioning is a lack of specific pharmacological tools: the independence of s-GABA_A_Rs gating from GABA binding makes impossible the use of competitive GABA antagonists for selective s-GABA_A_Rs silencing, whereas allosteric modulators such as benzodiazepines display a lack of specificity, tuning both GABA-dependent and GABA-independent effects (Bianchi and Macdonald, 2001; McCartney et al., 2007; Gerak, 2009).

**Figure 1:**
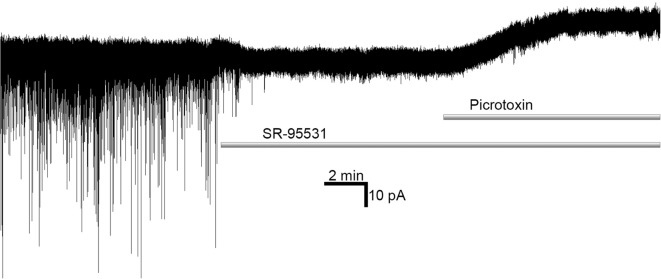
Competitive γ-aminobutyric acid (GABA) antagonist SR-95531 suppresses spontaneous GABA-ergic synaptic signaling, but does not affect tonic conductance; on the contrary, open-channel blocker picrotoxin applied after SR-95531 shuts spontaneously opening GABA-receptors (s-GABA_A_Rs), revealing the amount of inhibitory current passing through s-GABA_A_Rs independently of GABA binding.

Hence, to clarify the input of s-GABA_A_Rs into a given effect, differences in molecular mechanisms of SR- and PTX-induced GABA_A_Rs silencing have been used. SR is a competitive antagonist and thus negates GABA_A_R activity induced by GABA binding (i.e., it acts on conventional GABA_A_Rs); in contrast, PTX binds inside the GABA_A_R ion channel, and thus blocks all open channels, independently of the presence of GABA binding (i.e., it acts on both conventional GABA_A_Rs and s-GABA_A_Rs). Therefore, conventional GABA_A_R activity can be assessed as the change in the given effect obtained in the control vs. after application of SR, whereas s-GABA_A_R activity can be measured as the change in the effect obtained after SR application vs. after subsequent application of SR+PTX (Wlodarczyk et al., 2013)—see Figure 1. SR is a “silent” competitor for the GABA-binding site, i.e., it does not display inverse agonist properties. Obviously, competitive antagonists such as BIC, which display inverse agonism, cannot be used for the quantitative assessment of s-GABA_A_Rs effects: BIC was shown not only to suppress synaptic events as SR does but also to induce an outward shift of holding current (Wlodarczyk et al., 2013).

### s-GABARs Single-Channel Properties

The obvious step in the biophysical characterization of different subgroups of ionotropic receptors is a dissection of single-channel properties, such as electrical conductance, opening frequency and average open time. Single-channel recordings have repeatedly demonstrated similar or very close conductance values for s-GABA_A_Rs and conventional GABA_A_Rs (Mathers, 1985; Neelands et al., 1999; Birnir et al., 2000; O’Neill and Sylantyev, 2018a,b) thus making this parameter hardly applicable for distinguishing between two receptor subtypes. Similarly, the dependence of GABA_A_Rs opening frequency on the concentration of GABA, makes this parameter inapplicable for discrimination of effects of s-GABA_A_Rs and conventional GABA_A_Rs in single-channel recordings. In contrast, the average open time was found to be significantly lower for s-GABA_A_Rs than for conventional GABA_A_Rs. This generates a two-peak distribution of opening time values under physiological conditions when free GABA is present in extracellular space (O’Neill and Sylantyev, 2018a). Earlier observations demonstrated that the two-peak Gaussian distribution of average open times is a characteristic feature of GABA_A_Rs of at least three different subunit compositions (Mortensen et al., 2010). It is important to note that the mode values for shorter durations in that work were found to be similar, irrespective of the agonist’s type and concentration, thus representing an agonist-independent input. This suggests that: (i) s-GABA_A_Rs activity is a common element of integral GABA_A_R response; and (ii) that s-GABA_A_Rs represent a functionally similar receptor subgroup composed of receptors of various subunit compositions.

Another method of distinguishing between s-GABA_A_Rs and conventional GABA_A_Rs at a level of single-channel effects may potentially develop from the recent observation about the ability of benzodiazepine flurazepam to modulate GABA-dependent and GABA-independent GABA_A_R gating *via* different molecular mechanisms (Jatczak-Śliwa et al., 2018).

### s-GABARs Input Into Tonic Conductance

Overall, charge transfer with phasic events mediated by GABA_A_Rs (and induced by GABA binding) compared to that delivered by tonic conductance through GABA_A_Rs, displays a ratio of more than 9/1 (Cope et al., 2005; O’Neill and Sylantyev, 2018a). Taking into account that GABA-induced tonic current was found to be negligible under physiological concentrations of extracellular GABA, whereas under these conditions s-GABA_A_Rs generated a significant amount of tonic current (Wlodarczyk et al., 2013), s-GABA_A_Rs should be considered as a potential key element in the generation of lasting inhibitory tone and, in a wider context, in inter-neuronal crosstalk.

Tonic inhibition has been widely accepted to be a strong modulator of action potential (AP) generation (Hamann et al., 2002; Bonin et al., 2007), AP firing patterns (Häusser and Clark, 1997) and the coincidence detection time window for synaptic inputs (Tang et al., 2011). Experiments on s-GABA_A_Rs have readily confirmed their significant input into the regulation of the following phenomena: the modulation of AP generation (O’Neill and Sylantyev, 2018b), firing patterns (Botta et al., 2015; O’Neill and Sylantyev, 2018a), neurons’ rheobase, and the time window of coincidence detection of excitatory inputs (O’Neill and Sylantyev, 2018a).

### s-GABA_A_Rs Input Into Phasic Conductance

Several classical studies have demonstrated that GABA_A_Rs of specific subunit compositions (e.g., δ-GABA_A_Rs) which may be responsible for a lion’s share of tonic current (Nusser and Mody, 2002; Stell et al., 2003; Mortensen et al., 2010) are localized exclusively at the extrasynaptic membrane (Nusser et al., 1998; Wei et al., 2003). However, if s-GABA_A_Rs are a functionally similar group of receptors of different subunit composition (see “s-GABARs Single-Channel Properties” section), their absence in synapses would be highly doubtful. This, in turn, raises a question as to how (and whether) s-GABA_A_Rs modify synaptic (phasic) GABA-ergic inhibitory responses (inhibitory post-synaptic currents, IPSCs). In truth, recent studies have demonstrated their significant input into IPSC decay kinetics: s-GABA_A_Rs introduced a slow element of decay profile (O’Neill and Sylantyev, 2018a), probably due to their higher potency to GABA (Yeung et al., 2003) and/or modified receptor efficacy.

It was shown earlier that GABA_A_R-generated IPSC may contain fast and slow components with different sensitivities to GABA competitive antagonists, which resembles the functional profile of s-GABA_A_Rs (Kapur et al., 1997). In this research, the generation of fast and slow components of whole-cell IPSC was attributed to different cell regions: dendritic and somatic, respectively. On the other hand, later direct recordings of s-GABA_A_Rs activity confirmed a significant input of this receptor subtype into both whole-cell IPSCs (which are generated in synapses), and into IPSCs evoked in nucleated membrane patches, i.e., generated by GABA_A_Rs localized at a neural cell soma (O’Neill and Sylantyev, 2018a). On top of that, a significant input of δ-GABA_A_Rs into IPSCs was recently demonstrated (Sun et al., 2018), which confirms once again both the synaptic and extrasynaptic localization of GABA_A_Rs which display high tonic activity.

### Intracellular Regulatory Mechanisms of s-GABA_A_Rs Activity

The particular intracellular mechanisms which are used by neural cells to modulate the activity of GABA_A_Rs are still far from being completely understood; however, it has long been established that direct phosphorylation is of major importance (Brandon et al., 2002). It was shown that GABA_A_Rs functions can be modulated differentially (potentiated or suppressed) depending on the receptor subunit composition, the type of neuron, et cetera by cAMP-dependent protein kinase A (PKA), tyrosine kinase Src and PKC: refer to Brandon et al. (2002) for review. In particular, GABA_A_R-mediated tonic inhibitory currents were shown to be downregulated by PKC Bright and Smart, 2013, whereas PKA was found to enhance this type of inhibition (Carlson et al., 2016). In addition, GABA_A_Rs effects were repeatedly shown to be modulated by G-protein-coupled receptors *via* G-proteins of different types (Cai et al., 2002; Wang et al., 2002) which are, in turn, tightly connected to the regulation of PKC and PKA activity (Neves et al., 2002). Hence, the clarification of impact on s-GABA_A_Rs function delivered by intracellular regulatory factors (specifically, by various kinases and G-proteins), is one of the key steps needed for understanding and predicting s-GABA_A_Rs functional input into a neural transmission.

To date, there is little data on this. It has been demonstrated that in dentate gyrus granule cells of hippocampus PKC regulates tonic GABA-dependent inhibitory conductance but has no significant impact on the GABA-independent effects of s-GABA_A_Rs (O’Neill and Sylantyev, 2018b). However, at a longer time scale it was repeatedly shown that PKC and Ca^2+^/calmodulin-dependent protein kinase II increase tonic inhibition in hippocampus and amygdala due to enhanced phosphorylation and membrane insertion of β3-containing GABA_A_Rs (Saliba et al., 2012; Modgil et al., 2017) and α4-containing GABA_A_Rs; this PKC action can be potentiated by neurosteroids such as THDOC (Abramian et al., 2010, 2014; Romo-Parra et al., 2015). In turn, s-GABA_A_Rs-mediated tonic inhibition in dentate gyrus granule cells is controlled by G-proteins: non-specific block of G-proteins by pertussis toxin decreases the tonic current *via* the reduction of the s-GABA_A_Rs opening frequency (O’Neill and Sylantyev, 2018b).

In contrast to PKC, activation of PKA was found to increase the tonic current through α4β3δ and, to a lesser extent, α4β3γ2L-GABA_A_Rs in absence of GABA due to upregulation of single-channel opening frequency. Addition of GABA to an ambient solution, however, gradually decreased the sensitivity of GABA_A_Rs of both subunit compositions to modulation by PKA; such a modulation became insignificant when GABA concentration reached micromolar values (Tang et al., 2010).

It is important to note, however, that a significant part of GABA-independent s-GABA_A_Rs activity was found to be out of the control of any soluble cytoplasmic factors. GABA-independent openings of GABA_A_Rs were recorded from outside-out patches excised from dentate gyrus granule cells somata: in this preparation, all cytoplasmic signaling chains are surely destroyed (O’Neill and Sylantyev, 2018b). However, anchored kinases that modulate ionotropic receptors (Brandon et al., 2003; Carnegie and Scott, 2003) may still be responsible for at least a part of the s-GABA_A_Rs activity observed in outside-out patches.

## Conclusions and Further Research Directions

To date, there have been only a few publications highlighting the functional properties of s-GABA_A_Rs in living neurons. This imposes obvious limitations on conclusions in terms of the applicability for different brain regions and types of neurons. Nevertheless, the significant input of s-GABA_A_Rs into the modulation of output signal generation and into the integration of input signaling in a given neuron, suggests that s-GABA_A_R activity is one of the key actors that regulate neural inhibition.

Indeed, the relative importance of GABA-independent s-GABA_A_Rs signaling in a given region of the brain depends critically on the native concentration of GABA in the extracellular space. Different groups report *in vivo* concentrations varying by more than an order of magnitude: from less than 100 (Wlodarczyk et al., 2013) or 200 (Glaeser and Hare, 1975) nM to units of micromoles (Tossman et al., 1986; Takagi et al., 1993). Moreover, there may be local inhomogeneities of GABA concentrations due to cell-specific differences in the distribution and/or activity of GABA transporters and the elements of the GABA synthesis system. This was indirectly confirmed by the observation that the silencing of GAD-65 activity reduces tonic inhibitory currents in interneurons, but not in the pyramidal neurons of the hippocampal CA1 area (Song et al., 2011). A recent study on the hippocampus has demonstrated that at a GABA concentration of ~100 nM, the amount of GABA-induced tonic current (which can be suppressed by SR) is close to statistical noise (see example at Figure 1), and negligible when compared to that through GABA-independent openings of s-GABA_A_Rs (Wlodarczyk et al., 2013); on the contrary, SR has been shown to reveal a huge amount of tonic GABA-dependent current in thalamus (Cope et al., 2005). These data suggest that the relative impact of s-GABA_A_Rs into neural signaling varies widely, depending on the particular brain region and cell type. To the best of our knowledge, previous articles that discuss lower EC_50_ values (i.e., higher potency) of extrasynaptic GABA_A_Rs *in vivo* do not consider spontaneous channels and how they influence such measurements. This fact enforces the importance of the work on s-GABA_A_Rs pharmacology for an understanding of biophysical phenomena in living neurons.

The important question regarding s-GABA_A_Rs is whether or not these receptors represent a convergent group with similar functional properties, or if they share common receptor subunit(s). Numerous studies have attributed the majority (up to 75%) of GABA_A_R-delivered tonic inhibition to δ-containing GABA_A_Rs (Stell et al., 2003), which are abundant at extrasynaptic membranes (Nusser et al., 1998) but have been also found in synapses where they make a significant input into phasic inhibition (Sun et al., 2018), and in perisynaptic loci (Wei et al., 2003). The remaining portion of tonic inhibition is, to a large extent but not fully, produced by receptors containing the α5-subunit (Farrant and Nusser, 2005). Furthermore, the agonist-independent GABA_A_R openings were observed under similar conditions for receptors of three different subunit compositions (Mortensen et al., 2010). In addition, the observation that mutations in α1 and β2 subunits modulate spontaneous GABA_A_Rs gating (Baptista-Hon et al., 2017) prevents us from ruling out these subunits as potential alternative candidates to be involved in the formation of s-GABA_A_Rs. Combined with the facts of the GABA-independent tonic activity of α4-GABA_A_Rs (Tang et al., 2010) and spontaneous openings of α2β1ε-GABA_A_Rs which contribute to the baseline currents in whole-cell recordings (Wagner et al., 2005), the abovementioned data on GABA-independent activity suggest that GABA-independent inhibition is of poly-subtype origin, with a substantial part inherent in the non-δ- and non-α5-containing receptors.

In view of numerous subunits and subunit compositions of GABA_A_R which demonstrate spontaneous gating, the obvious question is: are there GABA_A_Rs subtype(s) which do not demonstrate GABA-independent activity? The existence of such GABA_A_Rs was suggested by the study showing that, in contrast to the α2α1ε receptor, responses of α2β1 and α2β1γ2-GABA_A_Rs do not produce a “baseline overshoot” associated with spontaneous openings (Wagner et al., 2005).

Therefore, data collected to date suggest revision of two traditional views, now common in fundamental neuroscience: (i) that tonic inhibitory conductance is generated by ambient GABA (due to proven significance of s-GABA_A_Rs input); and (ii) that tonic and phasic inhibition are mediated by different GABA_A_Rs subtypes (due to growing evidence that typical extrasynaptic GABA_A_Rs can make a significant contribution into IPSCs *via* a synaptic and/or perisynaptic presence).

It has been demonstrated that a scarcity of α1 subunit is correlated with resistance to anti-epileptic drugs (Bethmann et al., 2008), whereas increased α1-GABA_A_R expression in the hippocampus suppresses the development of temporal lobe epilepsy (TLE; Raol et al., 2006). Apart from that, it was shown that phasic GABA-ergic inhibition is lowered in TLE, whereas tonic GABA-ergic conductance remains intact (Palma et al., 2007; Pavlov et al., 2011), making tonic GABA-ergic current a perspective target for TLE treatment. The classical paradigm, where extracellular GABA triggers tonic GABA-ergic current, implies that the most effective therapeutic approach is to increase the concentration of GABA in the cerebrospinal fluid, and thus augment inhibitory conductance. However, this approach was repeatedly found to be ineffective (Cohen et al., 2002; Glykys et al., 2009) or even one that leads to epileptogenesis (Palma et al., 2006; Cope et al., 2009) due to various side effects. These side effects impose limitations on the clinical use of specific antiepileptic drugs that increase the concentration of GABA in cerebrospinal fluid (Sander and Hart, 1990; Leppik, 1995). In contrast, the modulation of s-GABA_A_Rs in GABA-independent manner promises an alternative for TLE treatment through the regulation of tonic conductance without the need to interfere with extracellular GABA concentration, thus avoiding the afore mentioned side effects.

Apart from the potential of α1-GABA_A_Rs for TLE treatment, α5-GABA_A_Rs (which also display GABA-independent activity) were found to be a perspective target for schizophrenia treatment (Lodge and Grace, 2011). Taking into account similar concentration of GABA found *in vivo* in the brains of schizophrenic patients and of a control group (Tayoshi et al., 2010), and the well-established fact that changes in tonic GABA-ergic inhibition are involved in the generation of schizophrenia symptoms (Damgaard et al., 2011), these data suggest a potentially important role of drugs targeting s-GABA_A_Rs in the suppression of schizophrenia development, since action through s-GABA_A_Rs in GABA-independent manner eliminates the need to modify GABA concentration in cerebrospinal fluid.

Another clinical implication of s-GABA_A_Rs rises from the fact that sedative and analgesic effects of gaboxadol (THIP) are mediated exclusively by α4-containing GABA_A_Rs (Chandra et al., 2006), that demonstrate GABA-independent activity.

## Author Contributions

NO and SS contributed to the conception and design of the article. NO received data displayed at the figure and analyzed literature connected to the topic, contributed to manuscript revision. SS wrote the manuscript.

## Conflict of Interest Statement

The authors declare that the research was conducted in the absence of any commercial or financial relationships that could be construed as a potential conflict of interest.

## References

[B1] Abramian A. M. Comenencia-Ortiz E. Vithlani M. Tretter E. V. Sieghart W. Davies P. A.. (2010). Protein kinase C phosphorylation regulates membrane insertion of GABA_A_ receptor subtypes that mediate tonic inhibition. J. Biol. Chem. 285, 41795–41805. 10.1074/jbc.m110.149229 20940303 PMC3009907

[B2] Abramian A. M. Comenencia-Ortiz E. Modgil A. Vien T. N. Nakamura Y. Moore Y. E.. (2014). Neurosteroids promote phosphorylation and membrane insertion of extrasynaptic GABA_A_ receptors. Proc. Natl. Acad. Sci. U S A 111, 7132–7137. 10.1073/pnas.1403285111 24778259 PMC4024867

[B3] Baptista-Hon D. T. Gulbinaite S. Hales T. G. (2017). Loop G in the GABA_A_ receptor α1 subunit influences gating efficacy. J. Physiol. 595, 1725–1741. 10.1113/jp273752 27981574 PMC5330932

[B4] Bethmann K. Fritschy J. M. Brandt C. Löscher W. (2008). Antiepileptic drug resistant rats differ from drug responsive rats in GABA_A_ receptor subunit expression in a model of temporal lobe epilepsy. Neurobiol. Dis. 31, 169–187. 10.1016/j.nbd.2008.01.005 18562204

[B5] Bianchi M. T. Macdonald R. L. (2001). Agonist trapping by GABA_A_ receptor channels. J. Neurosci. 21, 9083–9091. 10.1523/jneurosci.21-23-09083.2001 11717341 PMC6763914

[B6] Birnir B. Everitt A. B. Lim M. S. Gage P. W. (2000). Spontaneously opening GABA_A_ channels in CA1 pyramidal neurones of rat hippocampus. J. Membr. Biol. 174, 21–29. 10.1007/s002320001028 10741429

[B7] Bonin R. P. Martin L. J. MacDonald J. F. Orser B. A. (2007). α5GABA_A_ receptors regulate the intrinsic excitability of mouse hippocampal pyramidal neurons. J. Neurophysiol. 98, 2244–2254. 10.1152/jn.00482.2007 17715197

[B8] Botta P. Demmou L. Kasugai Y. Markovic M. Xu C. Fadok J. P.. (2015). Regulating anxiety with extrasynaptic inhibition. Nat. Neurosci. 18, 1493–1500. 10.1038/nn.4102 26322928 PMC4607767

[B9] Brandon N. Jovanovic J. Moss S. (2002). Multiple roles of protein kinases in the modulation of gamma-aminobutyric acid_(A)_ receptor function and cell surface expression. Pharmacol. Ther. 94, 113–122. 10.1016/s0163-7258(02)00175-4 12191597

[B10] Brandon N. J. Jovanovic J. N. Colledge M. Kittler J. T. Brandon J. M. Scott J. D. (2003). A-kinase anchoring protein 79/150 facilitates the phosphorylation of GABA_A_ receptors by cAMP-dependent protein kinase via selective interaction with receptor β subunits. Mole. Cell. Neurosci. 22, 87–97. 10.1016/s1044-7431(02)00017-9 12595241

[B11] Brickley S. G. Cull-Candy S. G. Farrant M. (1996). Development of a tonic form of synaptic inhibition in rat cerebellar granule cells resulting from persistent activation of GABA_A_ receptors. J Physiol. 497, 753–759. 10.1113/jphysiol.1996.sp021806 9003560 PMC1160971

[B12] Bright D. P. Smart T. G. (2013). Protein kinase C regulates tonic GABA_A_ receptor-mediated inhibition in the hippocampus and thalamus. Eur. J. Neurosci. 38, 3408–3423. 10.1111/ejn.12352 24102973 PMC4165308

[B13] Brown D. A. (1979). Extrasynaptic GABA systems. Trends Neurosci. 2, 271–273. 10.1016/0166-2236(79)90107-3

[B14] Cai X. Flores-Hernandez J. Feng J. Yan Z. (2002). Activity-dependent bidirectional regulation of GABA_A_ receptor channels by the 5-HT_(4)_ receptor-mediated signalling in rat prefrontal cortical pyramidal neurons. J. Physiol. 540, 743–759. 10.1113/jphysiol.2001.013391 11986365 PMC2290288

[B15] Carlson S. L. Bohnsack J. P. Patel V. Morrow A. L. (2016). Regulation of extrasynaptic GABA_A_ α4 receptors by ethanol-induced protein kinase, A, but not protein kinase C activation in cultured rat cerebral cortical neurons. J. Pharmacol. Exp. Ther. 356, 148–156. 10.1124/jpet.115.228056 26483396 PMC4702069

[B16] Carnegie G. K. Scott J. D. (2003). A-kinase anchoring proteins and neuronal signaling mechanisms. Genes Dev. 17, 1557–1568. 10.1101/gad.1095803 12842908

[B17] Chandra D. Jia F. Liang J. Peng Z. Suryanarayanan A. Werner D. F.. (2006). GABA_A_ receptor α4 subunits mediate extrasynaptic inhibition in thalamus and dentate gyrus and the action of gaboxadol. Proc. Natl. Acad. Sci. U S A 103, 15230–15235. 10.1073/pnas.0604304103 17005728 PMC1578762

[B18] Chiu C. S. Jensen K. Sokolova I. Wang D. Li M. Deshpande P.. (2002). Number, density and surface/cytoplasmic distribution of GABA transporters at presynaptic structures of knock-in mice carrying GABA transporter subtype 1-Green fluorescent protein fusions. J. Neurosci. 22, 10251–10266. 10.1523/jneurosci.22-23-10251.2002 12451126 PMC6758747

[B19] Cohen I. Navarro V. Clemenceau S. Baulac M. Miles R. (2002). On the origin of interictal activity in human temporal lobe epilepsy *in vitro*. Science 298, 1418–1421. 10.1126/science.1076510 12434059

[B20] Conti F. Minelli A. Melone M. (2004). GABA transporters in the mammalian cerebral cortex: localization, development and pathological implications. Brain Res. Rev. 45, 196–212. 10.1016/j.brainresrev.2004.03.003 15210304

[B21] Cope D. W. Hughes S. W. Crunelli V. (2005). GABA_A_ receptor-mediated tonic inhibition in thalamic neurons. J Neurosci. 25, 11553–11563. 10.1523/jneurosci.3362-05.2005 16354913 PMC6726040

[B22] Cope D. W. Di Giovanni G. Fyson S. J. Orbán G. Errington A. C. Lorincz M. L.. (2009). Enhanced tonic GABA_A_ inhibition in typical absence epilepsy. Nat. Med. 15, 1392–1398. 10.1038/nm.2058 19966779 PMC2824149

[B23] Damgaard T. Plath N. Neill J. C. Hansen S. L. (2011). Extrasynaptic GABA_A_ receptor activation reverses recognition memory deficits in an animal model of schizophrenia. Psychopharmacology 214, 403–413. 10.1007/s00213-010-2039-9 20957350

[B24] Farrant M. Nusser Z. (2005). Variations on an inhibitory theme: phasic and tonic activation of GABA_A_ receptors. Nat. Rev. Neurosci. 6, 215–229. 10.1038/nrn1625 15738957

[B25] Gerak L. R. (2009). Selective changes in sensitivity to benzodiazepines, and not other positive GABA_A_ modulators, in rats receiving flunitrazepam chronically. Psychopharmacology 204, 667–677. 10.1007/s00213-009-1497-4 19274455 PMC2965598

[B26] Glaeser B. S. Hare T. A. (1975). Measurement of GABA in human cerebrospinal fluid. Biochem. Med. 12, 274–282. 10.1016/0006-2944(75)90129-5 1137588

[B27] Glykys J. Mody I. (2007). Activation of GABA_A_ receptors: views from outside the synaptic cleft. Neuron 56, 763–770. 10.1016/j.neuron.2007.11.002 18054854

[B28] Glykys J. Dzhala V. I. Kuchibhotla K. V. Feng G. Kuner T. Augustine G.. (2009). Differences in cortical versus subcortical GABAergic signaling: a candidate mechanism of electroclinical uncoupling of neonatal seizures. Neuron 63, 657–672. 10.1016/j.neuron.2009.08.022 19755108 PMC2932871

[B29] Hamann M. Rossi D. J. Attwell D. (2002). Tonic and spillover inhibition of granule cells control information flow through cerebellar cortex. Neuron 33, 625–633. 10.1016/s0896-6273(02)00593-7 11856535

[B30] Häusser M. Clark B. A. (1997). Tonic synaptic inhibition modulates neuronal output pattern and spatiotemporal synaptic integration. Neuron 19, 665–678. 10.1016/s0896-6273(00)80379-7 9331356

[B31] Jatczak-Śliwa M. Terejko K. Brodzki M. Michałowski M. A. Czyzewska M. M. Nowicka J. M.. (2018). Distinct modulation of spontaneous and GABA-evoked gating by flurazepam shapes cross-talk between agonist-free and liganded GABA_A_ receptor activity. Front. Cell. Neurosci. 12:237. 10.3389/fncel.2018.00237 30210295 PMC6121034

[B32] Kapur A. Pearce R. A. Lytton W. W. Haberly L. B. (1997). GABA_A_-mediated IPSCs in piriform cortex have fast and slow components with different properties and locations on pyramidal cells. J. Neurophysiol. 78, 2531–2545. 10.1152/jn.1997.78.5.2531 9356403

[B33] Kozlov A. S. Angulo M. C. Audinat E. Charpak S. (2006). Target cell-specific modulation of neuronal activity by astrocytes. Proc. Natl. Acad. Sci. U S A 103, 10058–10063. 10.1073/pnas.0603741103 16782808 PMC1502505

[B34] Leppik I. E. (1995). Tiagabine: the safety landscape. Epilepsia 36, S10–S13. 10.1111/j.1528-1157.1995.tb06009.x 8595787

[B35] Lodge D. J. Grace A. A. (2011). Hippocampal dysregulation of dopamine system function and the pathophysiology of schizophrenia. Trends Pharmacol. Sci. 32, 507–513. 10.1016/j.tips.2011.05.001 21700346 PMC3159688

[B36] Maksay G. Thompson S. A. Wafford K. A. (2003). The pharmacology of spontaneously open α1 β3 ε GABA_A_ receptor-ionophores. Neuropharmacology 44, 994–1002. 10.1016/s0028-3908(03)00116-3 12763092

[B37] Mathers D. A. (1985). Spontaneous and GABA-induced single channel currents in cultured murine spinal cord neurons. Can. J. Physiol. Pharmacol. 63, 1228–1233. 10.1139/y85-203 2416415

[B38] McCartney M. R. Deeb T. Z. Henderson T. N. Hales T. G. (2007). Tonically active GABA_A_ receptors in hippocampal pyramidal neurons exhibit constitutive GABA-independent gating. Mol. Pharmacol. 71, 539–548. 10.1124/mol.106.028597 17090706

[B39] Miko A. Werby E. Sun H. Healey J. Zhang L. (2004). A TM2 residue in the β1 subunit determines spontaneous opening of homomeric and heteromeric γ-aminobutyric acid-gated ion channels. J. Biol. Chem. 279, 22833–22840. 10.1074/jbc.m402577200 15014066

[B40] Minelli A. DeBiasi S. Brecha N. C. Zuccarello L. V. Conti F. (1996). GAT-3, a high-affinity GABA plasma membrane transporter, is localized to astrocytic processes, and it is not confined to the vicinity of GABAergic synapses in the cerebral cortex. J. Neurosci. 16, 6255–6264. 10.1523/jneurosci.16-19-06255.1996 8815906 PMC6579190

[B41] Modgil A. Parakala M. L. Ackley M. A. Doherty J. J. Moss S. J. Davies P. A. (2017). Endogenous and synthetic neuroactive steroids evoke sustained increases in the efficacy of GABAergic inhibition via a protein kinase C-dependent mechanism. Neuropharmacology 113, 314–322. 10.1016/j.neuropharm.2016.10.010 27743930 PMC5148695

[B42] Mody I. Pearce R. A. (2004). Diversity of inhibitory neurotransmission through GABA_A_ receptors. Trends Neurosci. 27, 569–575. 10.1016/j.tins.2004.07.002 15331240

[B43] Mortensen M. Ebert B. Wafford K. Smart T. G. (2010). Distinct activities of GABA agonists at synaptic- and extrasynaptic-type GABA_A_ receptors. J. Physiol. 588, 1251–1268. 10.1113/jphysiol.2009.182444 20176630 PMC2872731

[B44] Neelands T. R. Fisher J. L. Bianchi M. Macdonald R. L. (1999). Spontaneous and γ-aminobutyric acid (GABA)-activated GABA_A_ receptor channels formed by ε subunit-containing isoforms. Mol. Pharmacol. 55, 168–178. 10.1124/mol.55.1.168 9882711

[B45] Neves S. R. Ram P. T. Iyengar R. (2002). G protein pathways. Science 296, 1636–1639. 10.1126/science.1071550 12040175

[B46] Nusser Z. Mody I. (2002). Selective modulation of tonic and phasic inhibitions in dentate gyrus granule cells. J. Neurophysiol. 87, 2624–2628. 10.1152/jn.2002.87.5.2624 11976398

[B47] Nusser Z. Sieghart W. Somogyi P. (1998). Segregation of different GABA_A_ receptors to synaptic and extrasynaptic membranes of cerebellar granule cells. J. Neurosci. 18, 1693–1703. 10.1523/jneurosci.18-05-01693.1998 9464994 PMC6792611

[B48] O’Neill N. Sylantyev S. (2018a). Spontaneously opening GABA_A_ receptors play a significant role in neuronal signal filtering and integration. Cell Death Dis. 9:813. 10.1038/s41419-018-0856-7 30042389 PMC6057890

[B49] O’Neill N. Sylantyev S. (2018b). Feature Article: selective modulation of tonically active GABA_A_ receptor functional subgroups by G-proteins and protein kinase C. Exp. Biol. Med. 243, 1046–1055. 10.1177/1535370218800980 30205722 PMC6434461

[B50] Oláh S. Füle M. Komlósi G. Varga C. Báldi R. Barzó P.. (2009). Regulation of cortical microcircuits by unitary GABA-mediated volume transmission. Nature 461, 1278–1281. 10.1038/nature08503 19865171 PMC2771344

[B51] Otis T. S. Staley K. J. Mody I. (1991). Perpetual inhibitory activity in mammalian brain slices generated by spontaneous GABA release. Brain Res. 545, 142–150. 10.1016/0006-8993(91)91280-e 1650273

[B52] Palma E. Amici M. Sobrero F. Spinelli G. Di Angelantonio S. Ragozzino D.. (2006). Anomalous levels of Cl- transporters in the hippocampal subiculum from temporal lobe epilepsy patients make GABA excitatory. Proc. Natl. Acad. Sci. U S A 103, 8465–8468. 10.1073/pnas.0602979103 16709666 PMC1482515

[B53] Palma E. Roseti C. Maiolino F. Fucile S. Martinello K. Mazzuferi M.. (2007). GABA_A_-current rundown of temporal lobe epilepsy is associated with repetitive activation of GABA_A_ “phasic” receptors. Proc. Natl. Acad. Sci. U S A 104, 20944–20948. 10.1073/pnas.0710522105 18083839 PMC2409246

[B54] Pavlov I. Huusko N. Drexel M. Kirchmair E. Sperk G. Pitkänen A.. (2011). Progressive loss of phasic, but not tonic, GABA_A_ receptor-mediated inhibition in dentate granule cells in a model of post-traumatic epilepsy in rats. Neuroscience 194, 208–219. 10.1016/j.neuroscience.2011.07.074 21840377

[B55] Raol Y. H. Lund I. V. Bandyopadhyay S. Zhang G. Roberts D. S. Wolfe J. H.. (2006). Enhancing GABA_A_ receptor alpha 1 subunit levels in hippocampal dentate gyrus inhibits epilepsy development in an animal model of temporal lobe epilepsy. J. Neurosci. 26, 11342–11346. 10.1523/jneurosci.3329-06.2006 17079662 PMC6674546

[B56] Romo-Parra H. Blaesse P. Sosulina L. Pape H. C. (2015). Neurosteroids increase tonic GABAergic inhibition in the lateral section of the central amygdala in mice. J. Neurophysiol. 113, 3421–3431. 10.1152/jn.00045.2015 25787948 PMC4455571

[B57] Saliba R. S. Kretschmannova K. Moss S. J. (2012). Activity-dependent phosphorylation of GABA_A_ receptors regulates receptor insertion and tonic current. EMBO J. 31, 2937–2951. 10.1038/emboj.2012.109 22531784 PMC3395084

[B58] Sander J. W. Hart Y. M. (1990). Vigabatrin and behaviour disturbances. Lancet 335:57. 10.1016/0140-6736(90)90190-g 1967367

[B59] Song I. Savtchenko L. Semyanov A. (2011). Tonic excitation or inhibition is set by GABA_A_ conductance in hippocampal interneurons. Nat. Commun. 2:376. 10.1038/ncomms1377 21730957 PMC3144593

[B60] Stell B. M. Brickley S. G. Tang C. Y. Farrant M. Mody I. (2003). Neuroactive steroids reduce neuronal excitability by selectively enhancing tonic inhibition mediated by delta subunit-containing GABA_A_ receptors. Proc. Natl. Acad. Sci. U S A 100, 14439–14444. 10.1073/pnas.2435457100 14623958 PMC283610

[B61] Sun M. Y. Shu H. J. Benz A. Bracamontes J. Akk G. Zorumski C. F.. (2018). Chemogenetic isolation reveals synaptic contribution of δ GABA_A_ receptors in mouse dentate granule neurons. J. Neurosci. 38, 8128–8145. 10.1523/jneurosci.0799-18.2018 30076210 PMC6146493

[B62] Takagi K. Ginsberg M. D. Globus M. Y. Dietrich W. D. Martinez E. Kraydieh S.. (1993). Changes in amino acid neurotransmitters and cerebral blood flow in the ischemic penumbral region following middle cerebral artery occlusion in the rat: correlation with histopathology. J. Cereb. Blood Flow Metab. 13, 575–585. 10.1038/jcbfm.1993.75 8100237

[B63] Tang X. Hernandez C. C. Macdonald R. L. (2010). Modulation of spontaneous and GABA-evoked tonic α4β3δ and α4β3γ2L GABA_A_ receptor currents by protein kinase A. J. Neurophysiol. 103, 1007–1019. 10.1152/jn.00801.2009 19939957 PMC2822691

[B64] Tang Z. Q. Dinh E. H. Shi W. Lu Y. (2011). Ambient GABA-activated tonic inhibition sharpens auditory coincidence detection via a depolarizing shunting mechanism. J. Neurosci. 31, 6121–6131. 10.1523/jneurosci.4733-10.2011 21508237 PMC3090224

[B65] Tayoshi S. Nakataki M. Sumitani S. Taniguchi K. Shibuya-Tayoshi S. Numata S.. (2010). GABA concentration in schizophrenia patients and the effects of antipsychotic medication: A proton magnetic resonance spectroscopy study. Schizophr. Res. 117, 83–91. 10.1016/j.schres.2009.11.011 20022731

[B66] Tossman U. Jonsson G. Ungerstedt U. (1986). Regional distribution and extracellular levels of amino acids in rat central nervous system. Acta Physiol. Scand. 127, 533–545. 10.1111/j.1748-1716.1986.tb07938.x 2875604

[B67] Wagner D. A. Goldschen-Ohm M. P. Hales T. G. Jones M. V. (2005). Kinetics and spontaneous open probability conferred by the ε subunit of the GABA_A_ receptor. J. Neurosci. 25, 10462–10468. 10.1523/jneurosci.1658-05.2005 16280584 PMC6725813

[B68] Wang X. Zhong P. Yan Z. (2002). Dopamine D4 receptors modulate GABAergic signaling in pyramidal neurons of prefrontal cortex. J. Neurosci. 22, 9185–9193. 10.1523/jneurosci.22-21-09185.2002 12417643 PMC6758062

[B69] Wei W. Zhang N. Peng Z. Houser C. R. Mody I. (2003). Perisynaptic localization of delta subunit-containing GABA_A_ receptors and their activation by GABA spillover in the mouse dentate gyrus. J. Neurosci. 23, 10650–10661. 10.1523/jneurosci.23-33-10650.2003 14627650 PMC6740905

[B70] Wlodarczyk A. I. Sylantyev S. Herd M. B. Kersanté F. Lambert J. J. Rusakov D. A.. (2013). GABA-independent GABA_A_ receptor openings maintain tonic currents. J. Neurosci. 33, 3905–3914. 10.1523/jneurosci.4193-12.2013 23447601 PMC3591781

[B71] Yeung J. Y. Canning K. J. Zhu G. Pennefather P. MacDonald J. F. Orser B. A. (2003). Tonically activated GABA_A_ receptors in hippocampal neurons are high-affinity, low-conductance sensors for extracellular GABA. Mol. Pharmacol. 63, 2–8. 10.1124/mol.63.1.2 12488530

